# The common homocystinuria-associated P1173L variant of human methionine synthase impairs reductive methylation

**DOI:** 10.1016/j.jbc.2025.110366

**Published:** 2025-06-11

**Authors:** Arkajit Guha, Ruma Banerjee

**Affiliations:** Department of Biological Chemistry, Michigan Medicine, University of Michigan, Ann Arbor, Michigan, USA

## Abstract

Human methionine synthase (MTR) is a cobalamin-dependent enzyme that catalyzes the transfer of a methyl group from methyltetrahydrofolate to homocysteine to form methionine and tetrahydrofolate. The enzyme is susceptible to oxidative inactivation and is repaired by methionine synthase reductase (MTRR) in the presence of NADPH and S-adenosylmethionine (AdoMet). The P1173L missense mutation is the most common clinical variant of MTR and causes homocystinuria, an inborn error of metabolism that is associated with aggressive occlusive cardiovascular diseases. In this study, we report that co-expression of MTR with the B_12_ chaperone MMADHC helps overcome prior challenges with soluble expression of full-length human MTR. Kinetic analysis reveals that wild-type and P1173L MTR exhibit comparable specific activities in the *in vitro* assay with an artificial reducing system although the *K*_M_ for AdoMet is ∼40-fold higher for the variant. In contrast, the P1173L variant is ∼30-fold less active in the presence of the physiologically relevant MTRR/NADPH-reducing system. EPR and kinetic analyses reveal that complex formation with MTRR, which limits the reactivation reaction in wild-type MTR, is unaffected in P1173L MTR, pointing to a switch in the rate-limiting step. Pre-steady state kinetic studies reveal pleiotropic impacts of the P1173L mutation, with electron transfer from MTRR to cob(II)alamin being rate-liming. Our study predicts that physiologically relevant small-molecule electron donors, some of which have been tested in this study, might have therapeutic potential to circumvent the penalties associated with P1173L MTR.

Vitamin B_12_ or cobalamin is an essential organometallic cofactor that is obtained from the diet and required by two human enzymes, methylcobalamin (MeCbl)-dependent methionine synthase (MTR) in the cytoplasm and 5′deoxyadenosylcobalamin-dependent methylmalonyl CoA mutase in the mitochondrion (1). These B_12_ enzymes are supported by a trafficking pathway comprising chaperones, which were discovered by clinical genetics studies on patients with inborn errors of B_12_ metabolism; the chaperones are key to ensuring fidelity and minimizing spurious reactivity during the cofactor loading process (2, 3, 4). Defects in B_12_ trafficking pathway proteins lead to combined or isolated homocystinuria and methylmalonic aciduria depending on whether the affected locus is in the common or cytoplasmic *versus* mitochondrial branches of the pathway. Studies over the past decade have provided mechanistic insights into chaperone functions and defects associated with variants in the mitochondrial branch (5, 6, 7, 8, 9, 10). By comparison, the proteins in the cytoplasmic branch have been less well characterized, leading to a paucity of mechanistic insights into the associated biochemical penalties.

Several B_12_-binding proteins ferry the cofactor from the diet to the cell surface and from there, into the cytoplasm (11). Both B_12_ enzymes rely on the cytoplasmic chaperones MMACHC and MMADHC. While MMACHC processes dietary cobalamins with varying upper axial ligands to a common cob(II)alamin intermediate (12, 13, 14, 15), MMADHC interacts with both MMACHC (16) and MTR (17) in the cytoplasmic branch; its role in the mitochondrial branch remains elusive. MMADHC has an intrinsically disordered N-terminal third and a well-folded core (18) that binds aquocobalamin (OH_2_-Cbl) from solution (Fig. 1*A*) or coordinates cob(II)alamin bound to MMACHC, both *via* a cobalt-sulfur (Co-S) bond at Cys-261 (16). Mutations in the N and C-terminal regions of MMADHC lead to isolated homocystinuria and methylmalonic aciduria, respectively, while mutations in the central region lead to combined disease (19).

**Figure 1:**
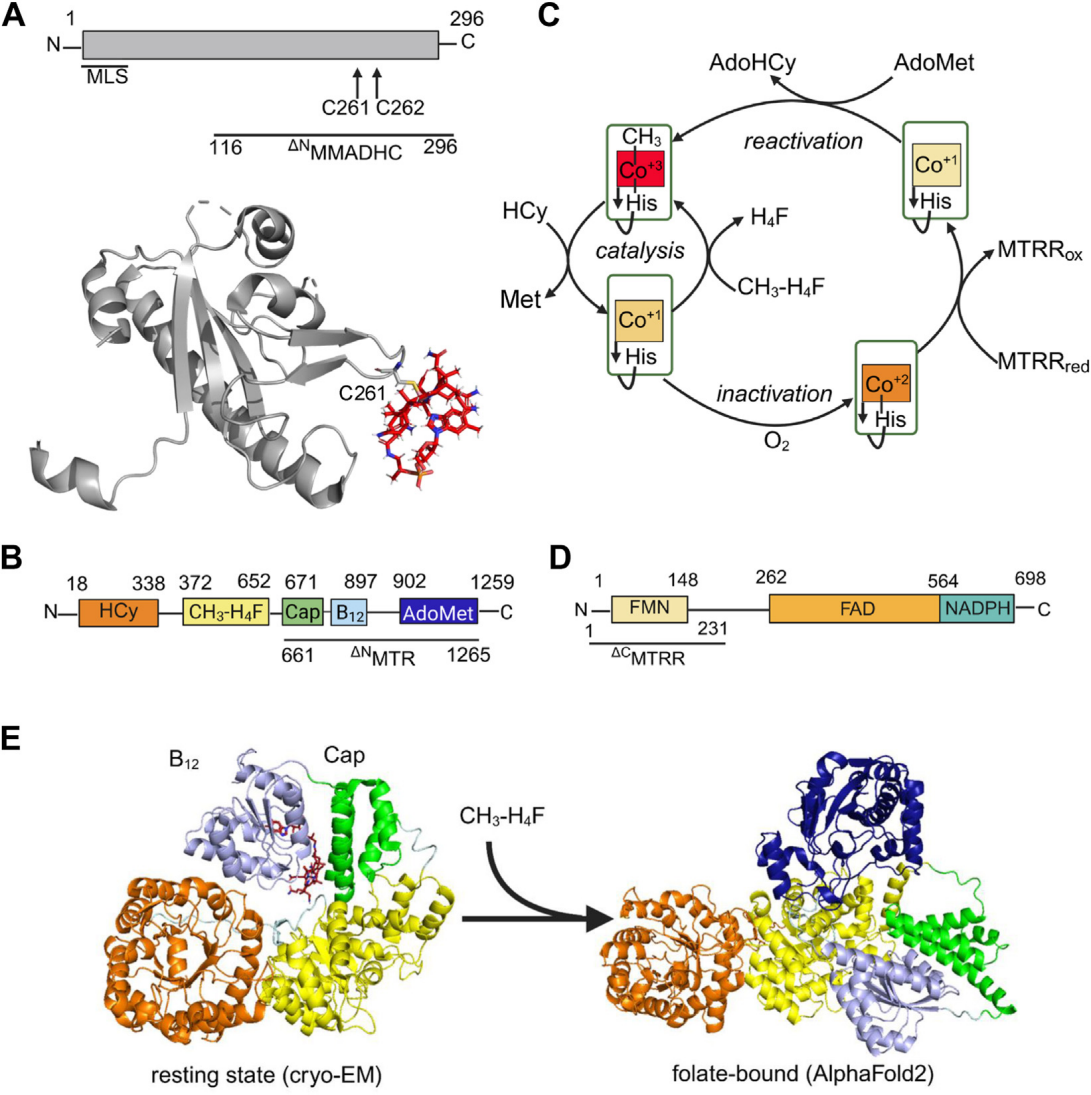
**Structure and reactions catalyzed by MMADHC, MTR, and MTRR.***A*, cartoon of full-length and MMADHC where MLS refers to the mitochondrial leader sequence (*top*), and structure of ^ΔN^MMADHC (PDB: 6X8Z). Cys-261 in the β-hairpin provides the sulfur ligand to the B_12_ (*red sticks*). *B*, domain organization of MTR and boundaries of ^ΔN^MTR used in this study. *C*, the MTR catalytic and the MTRR-dependent reactivation cycles. MTR catalyzes two methyl transfer reactions where the methyl group from 5-methyltetrahydrofolate (CH_3_-H_4_F) is transferred to homocysteine (HCy) to form H_4_F and methionine respectively while the enzyme cycles between methylcobalamin (MeCbl) and cob(I)alamin states. When cob(I)alamin is accidentally oxidized to the inactive cob(II)alamin, methionine synthase reductase (MTRR) catalyzes an NADPH/AdoMet-dependent reductive methylation to reactivate MTR. *D*, Domain organization of MTRR and boundaries of ^ΔC^MTRR used in this study. *E*, cryo-EM and AlphaFold2 analysis of a bacterial MTR predicts large conformational changes associated with CH_3_-H_4_F binding, which allows stabilization of conformations where the Cap domain disengages and exposes the B_12_ for reactivity. The AdoMet domain is disordered and missing in the cryoEM structure on the left.

Human MTR is a 140.5 kDa multidomain protein (Fig. 1*B*) that converts methyltetrahydrofolate (CH_3_-H_4_F) and homocysteine to tetrahydrofolate (H_4_F) and methionine (Fig. 1*C*), respectively (20). During catalysis, cobalamin cycles between the methylcobalamin (MeCbl) and cob(I)alamin states. The *Escherichia coli* MTR is estimated to inactivate once every ∼2000 turnovers, forming cob(II)alamin (Fig. 1*C*) (21). Methionine synthase reductase (MTRR), a dedicated redox partner of MTR (22, 23), is a diflavin oxidoreductase that binds flavin mononucleotide, flavin adenine dinucleotide and NADPH in distinct domains (24, 25, 26) (Fig. 1*D*). The flavin cofactors on MTRR mediate one-electron transfer from NADPH to cob(II)alamin on MTR, forming cob(I)alamin, which is subsequently methylated by S-adenosylmethionine (AdoMet) (26, 27). Following MeCbl formation in the repair cycle, MTR reenters the catalytic cycle (Fig. 1*C*).

Structural studies on bacterial MTR have revealed that in its resting state, the capped B_12_ domain is nestled between the homocysteine and CH_3_-H_4_F domains, while the AdoMet domain is conformationally dynamic (Fig. 1*E*) (28). CH_3_-H_4_F binding is predicted to lead to a conformational change that disengages the cap from the B_12_ domain, exposing the cofactor to the substrate domains (Fig. 1*E*). During reactivation, the AdoMet domain interacts with the B_12_ domain in a conformation that protects B_12_ from solvent exposure, while the cap is disengaged (29). In this conformation, MTR is also predicted to interact with MTRR.

MTR straddles two major metabolic pathways, the folate and methionine cycles. As the only enzyme in mammals that converts CH_3_-H_4_F to H_4_F, MTR is central to freeing up the cellular folate pool for nucleotide biosynthesis (20). Simultaneously, the generation of methionine by MTR recycles this essential amino acid for use in protein synthesis and as a substrate for AdoMet, a major methyl group donor. Mutations in the MTR gene are associated with megaloblastic anemia, hypomethionemia, and homocystinuria; the last is characterized by increased blood homocysteine levels and attendant aggressive, occlusive cardiovascular diseases (30, 31). Despite the high sequence similarity between the well-studied *E. coli* and human MTRs (53% sequence identity), characterization of the human enzyme has been thwarted by poor soluble expression of the full-length recombinant protein (32). This, in turn, has hampered studies on the biochemical penalties associated with clinical variants of MTR.

In this study, we report a solution to the recombinant full-length MTR expression problem by co-expression with the B_12_ chaperone MMADHC, which has enabled kinetic characterization of the wild-type protein. Furthermore, we have also elucidated how the common P1173L disease-causing variant reported in ∼70% of MTR patients (33), compromises function. The P1173L mutation is usually found in a compound heterozygous state, *i.e.*, is present on one allele with a different mutation on the second allele. Most patients with the P1173L mutation show early onset of symptoms, *i.e.*, in the first year of life although in other individuals, the age of presentation is significantly later (2–38 years), depending on the nature of the second mutation. Pro-1173 is highly conserved and located in a proline-rich loop in the AdoMet domain (34). It is flanked by residues that are involved in direct interactions with AdoMet (35). Our study reveals that the specific activities of wild-type and P1173L MTR are similar in the *in vitro* assay, which employs an artificial reducing system, although the *K*_M_ for AdoMet is ∼40-fold higher in the variant. In the presence of the physiological MTRR/NADPH reducing system, the specific activity of P1173L MTR is decreased ∼30-fold, thus isolating the biochemical penalty to the reductive activation cycle. Detailed pre-steady state kinetics analysis reveals multiple impacts of the P1173L mutation, including impaired electron transfer from MTRR, cob(I)alamin methylation by AdoMet, and a final conformational change gating entry into the catalytic cycle. Protein flexibility combined with AlphaFold2 analysis predicts that the P1173L mutation decreases flexibility on the face of the AdoMet domain that interacts with MTRR, providing a structural framework for interpreting the rate-limiting electron transfer to cob(II)alamin-bound MTR.

## Results

### Purification of full-length human MTR

**Figure 2:**
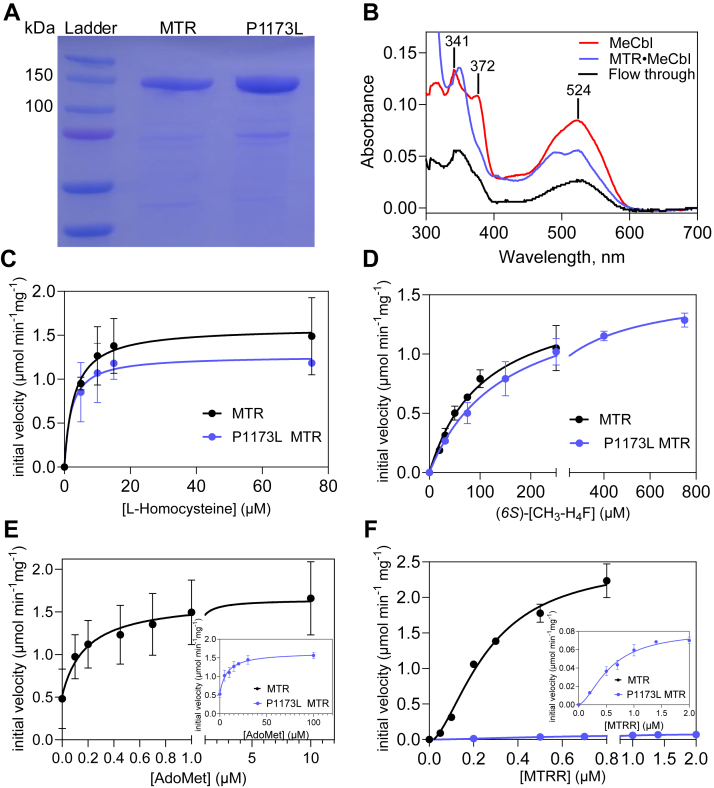
**Purification and kinetic characterization of full-length recombinant human MTR.***A*, SDS-PAGE analysis of wild-type and P1173L MTR (140.5 KDa) after a two-step purification. 5 μg of protein was loaded per lane. *B*, MeCbl (10 μM, *red*) was mixed with MTR (20 μM) in Buffer B and incubated at 20 °C for 30 min; followed by centrifugation through a Nanosep column. Following centrifugation, the spectrum of the filtrate (*blue*) *versus* the flowthrough (*black*) indicated that MeCbl was largely bound to MTR. *C*–*F*, dependence of wild-type MTR (*black*) and P1173L MTR (*blue*) activity on the concentrations of L-homocysteine (C), (*6S*)*-*CH_3_-H_4_F (*D*), AdoMet (*E*) and MTRR (*F*). The data represent the mean ± SD of three independent experiments.

While the sequence homology between *E. coli* and human MTR is high, sustained efforts to express recombinant full-length MTR have been challenging due in part, to its instability (32, 36). A serendipitous finding that OH_2_-Cbl binding to MMADHC is precluded when it is pre-mixed with ^ΔN^MTR, indicated that the apo-forms of these two proteins interact (17), and suggested that co-expression might help stabilize MTR. Indeed, co-expression of full-length MTR (6x-His tagged) and ^ΔN^MMADHC (GST-tagged) followed by Ni-NTA affinity and size exclusion chromatography, yielded ∼3 mg MTR per liter of culture, which was judged to be ∼95% pure by SDS PAGE analysis (Fig. 2*A*).

### Steady state kinetic characterization of wild-type MTR

Recombinant MTR bound MeCbl from solution (Fig. 2*B*), indicating that it is properly folded. The standard *in vitro* assay in which reducing equivalents are provided by a chemical (DTT/H_2_OCbl) or physiological (MTRR/NADPH) reducing system (32), yielded specific activities of 1.5 ± 0.1 μmol min^−1^ mg^−1^ and 2.5 ± 0.4 μmol min^−1^ mg^−1^ at 37 °C, respectively. Michaelis Menten kinetic analysis yielded *K*_M_ values of 3 ± 2 μM for L-homocysteine, 108 ± 18 μM for (*6S*)-CH_3_-H_4_F and 190 ± 130 nM for AdoMet (Fig. 2, *C*–*E*, Table 1). From the sigmoidal dependence of MTR activity on MTRR concentration, a *K*_*act*_ of 260 ± 46 nM was estimated (Fig. 2*F*, Table 1).

**Table 1:** Comparison of steady-state kinetic parameters for wild-type and P1173L MTR^a^

MTR	Specific activity (μmol min^−1^ mg^−1^)	*K*_M(Hcy)_^b^ μM	*K*_M(CH3-H4F)_^b^ μM	*K*_M(AdoMet)_ μM	*K*_act(MTRR)_ nM
Wild-type	1.5 ± 0.1 (DTT/H_2_OCbl) 2.5 ± 0.4 (MTRR/NADPH)	3 ± 2	108 ± 18	0.19 ± 0.13	260 ± 46
P1173L	1.5 ± 0.2 (DTT/H_2_OCbl) 0.08 ± 0.007 (MTRR/NADPH)	2 ± 1	147 ± 18	8 ± 2	580 ± 80

a The data represent mean ± SD from three independent experiments.

b The values are reported for the L-homocysteine and (*6S*)-CH_3_-H_4_F isomers.

### CH_3_-H_4_F promotes AdoMet-dependent MeCbl synthesis during cofactor loading

**Figure 3:**
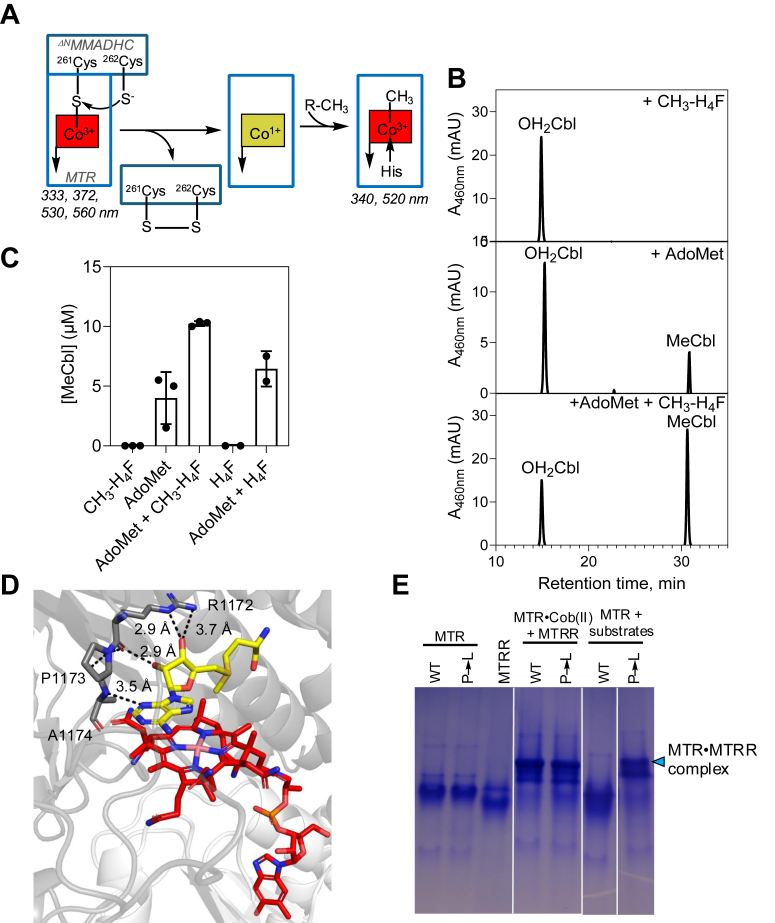
**AdoMet is the methyl donor to B_12_ following cofactor loading and defect associated with P1173L MTR in the reactivation cycle.***A*, postulated mechanism of B_12_ transfer from ^ΔN^MMADHC to MTR. R-CH_3_ represents the methyl donor to the B_12_ on MTR, which could in principle, be AdoMet or CH_3_-H_4_F. *B*, mixing ^ΔN^MMADHC-S-Cbl (25 μM) with MTR (50 μM), which had been pre-incubated with 250 μM CH_3_-H_4_F (*top*), 250 μM AdoMet (*middle*) and 250 μM each of CH_3_-H_4_F and AdoMet (*bottom*) for 60 min under anaerobic conditions led to MeCbl formation only when AdoMet was present. *C*, quantitation of MeCbl formed (mean ± SD) in (*B*). *D*, alphaFold model of human MTR showing interactions between the flanking R1172 and A1174 residues and AdoMet (*yellow sticks*). B_12_ is shown in *red sticks*. *E*, native gel analysis shows that cob(II)alamin-bound wild-type (WT) or P1173L (P→L) MTR form a complex with MTRR (*blue arrowhead*), which is stable when P1173L but not wild-type MTR is active in the presence of substrates (CH_3_-H_4_F, homocysteine, AdoMet and NADPH). Lanes were spliced from the same gel for representation purposes as indicated by solid white lines. The data are representative of three independent experiments.

Cofactor loading from MMADHC to MTR involves nucleophilic displacement of the Co-S bond by Cys-262, forming cob(I)alamin and a cysteine disulfide (Fig. 3*A*). Since cob(I)alamin is a common intermediate that is methylated by CH_3_-H_4_F in the catalytic, but AdoMet in the repair cycle (Fig. 1*C*), we asked which of the two donors is involved in the cofactor loading process.

In the absence of either methyl donor, or in the presence of CH_3_-H_4_F alone, a single peak corresponding to OH_2_Cbl (retention time ∼15 min) was observed by HPLC (Fig. 3*B*). H_2_OCbl is formed by cob(I)alamin oxidation and the lack of MeCbl in the presence of CH_3_-H_4_F rules out its use as a methyl donor during the loading process. In the presence of AdoMet alone, a peak corresponding to MeCbl (retention time ∼30 min) was seen (Fig. 3*B*) whose intensity increased when both methyl donors were present (Fig. 3*C*). Interestingly, H_4_F also increased AdoCbl-dependent MeCbl synthesis albeit to a lower extent than CH_3_-H_4_F (Fig. 3*C*). These data confirm that AdoMet serves as the methyl donor to cob(I)alamin during cofactor loading, while the presence of either CH_3_-H_4_F or H_4_F promotes the methylation reaction.

### The P1173L mutation impairs MTR reactivation

To understand the biochemical penalty associated with the P1173L mutation, the recombinant variant was purified and obtained in similar yield and comparable purity as wild-type MTR (Fig. 2*A*). Pro-1173 resides in a proline-rich loop and is flanked by Arg-1172 and Ala-1174, which are predicted to interact with AdoMet in the AlphaFold model (37) of human MTR (Fig. 3*D*). Its location in the regulatory domain suggests that the P1173L mutation is likely to impair AdoMet-dependent repair rather than the catalytic cycle.

When inactive cob(II)alamin-bound MTR was mixed with MTRR, a stable interprotein complex was seen by native PAGE analysis (Fig. 3*E*). However, when catalytically active and MeCbl-loaded MTR was mixed with substrates (*i.e.* homocysteine, CH_3_-H_4_F, AdoMet, MTRR, and NADPH), a stable interprotein complex with MTRR was seen with P1173L but not wild-type MTR (Fig. 3*E*). These data suggest that the P1173L mutation affects one or more steps in the repair cycle, impairing the reentry of MTR to the catalytic cycle.

The specific activity of P1173L MTR was ∼30-fold lower with the physiological MTRR/NADPH (0.08 ± 0.007 μmol min^−1^ mg^−1^) but indistinguishable from wild-type MTR with the chemical DTT/B_12_ (1.5 ± 0.2 μmol min^−1^ mg^−1^) reducing system (Table 1). Furthermore, the *K*_M_ for AdoMet (8 ± 2 μM) was ∼40-fold higher and the *K*_act_ for MTRR (580 ± 80 nM) was ∼2-fold higher for P1173L *versus* wild-type MTR (Fig. 2, *E* and *F*). In contrast, the *K*_M_ values for L-homocysteine (2 ± 1 μM) and (*6S*)-CH_3_-H_4_F (147 ± 18 μM) were virtually unaffected by the mutation (Fig. 2, *C* and *D*, Table 1). Collectively, the kinetic and native gel data help localize the penalty incurred by the P1173L mutation to the reactivation cycle.

Since the artificial DTT/B_12_ reducing system masked the penalty associated with the P1173L mutation, we tested whether physiologically relevant small molecule electron donors can substitute for DTT. While ascorbate, dihydrolipoic acid (DHLA), and ascorbate/B_12_ did not support MTR activity, DHLA/B_12_ did. The specific activity of wild-type (1.1 μmol min^−1^ mg^−1^) was similar while that of P1173L (0.7 μmol min^−1^ mg^−1^) MTR was two-fold lower with DHLA/B_12_ than with DTT/B_12_. These data suggest that a small molecule electron donor such as DHLA might have therapeutic potential for overcoming the biochemical penalty incurred by the common P1173L mutation in MTR.

### Pre-steady state kinetics analysis of the reactivation reaction

**Figure 4:**
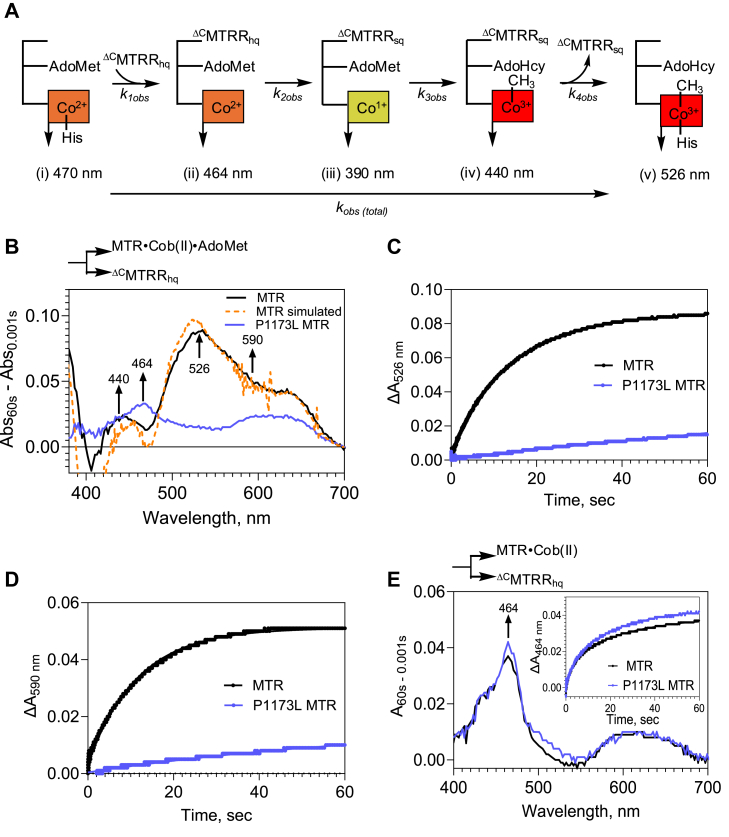
**The P1173L mutation impairs the reductive methylation of MTR.***A*, scheme showing the spectrally distinct steps involved in MTR repair. *B*, Cob(II)alamin-bound wild-type or P1173L MTR (40 μM) pre-incubated with AdoMet (400 μM) was rapidly mixed with 70 μM ^ΔC^MTRR_hq_ in an anaerobic stopped flow spectrophotometer. Difference spectra obtained at 60 s with wild-type (*black trace*) and 200 s with P1173L (*blue trace*) are shown and the simulated spectral changes for wild-type MTR (*orange trace*). *C–D*, time-dependent change in absorbance at 526 nm (*C*) and 590 nm (*D*) for wild-type (*black*) and P1173L (*blue*) MTR. *E*, Cob(II)alamin-bound wild-type or P1173L MTR (40 μM) was rapidly mixed with ^ΔC^MTRR_hq_ (70 μM) and spectra were monitored for 60 s. Difference spectra for wild-type (*black*) and P1173L (*blue*) MTR show an increase at 464 nm, corresponding to complex formation between the two proteins. *Inset*. The kinetics of complex formation were similar for both enzymes. The data are representative of three independent experiments.

Next, we used pre-steady state kinetic analysis to dissect the steps in the reactivation cycle that are impacted by the P1173L mutation. MTRR is a di-flavin oxidoreductase in which electrons are transferred from NADPH successively through FAD and FMN to MTR (24, 26). We expressed the N-terminal FMN-binding domain of MTRR (^ΔC^MTRR, Fig. 1*D*) to minimize spectral contributions from the C-terminal FAD-binding domain. In analogy with the reductive methylation mechanism described for *E. coli* MTR (38), the first step is predicted to involve complex formation between human MTR•cob(II)alamin and the FMN hydroquinone of ^ΔC^MTRR (^ΔC^MTRR_hq_) and is accompanied by loss of the conserved His-785 coordination to the cobalt ion (Fig. 4*A*, i→ii). Loss of the axial nitrogen ligand favors the reduction of cob(II)alamin to cob(I)alamin, which is rapidly methylated by AdoMet to form 5-coordinate (5-c) MeCbl (39, 40). In the final step, the FMN semiquinone form of ^ΔC^MTRR (^ΔC^MTRR_sq_) dissociates, which is accompanied by re-engagement of the His-785 ligand, forming MTR-bound 6-coordinate (6-c) MeCbl, which is poised to enter the catalytic cycle.

We determined the rate for the reductive methylation reaction (Fig. 4A, i→v) by rapidly mixing ^ΔC^MTRR_hq_ (70 μM) with wild-type MTR•cob(II)alamin (40 μM) in the presence of a 10-fold excess AdoMet in an anaerobic stopped-flow spectrophotometer. Difference spectra (recorded between 1 msec to 60 s) revealed an increase in absorbance at 440 and 526 nm corresponding to the formation of 5-c and 6-c MeCbl, respectively (Fig. 4B, *black trace*). A concomitant increase at 590 nm was assigned to the formation of ^ΔC^MTRR_sq_. The difference spectrum was deconvoluted by analysis of the component spectra of the B_12_ and ^ΔC^MTRR species (Fig. S1, *A* and *B*), which allowed estimation of the difference extinction coefficients at 526 nm for 6-c MeCbl formation (Δε_526_ = 8 mM^−1^cm^−1^), 440 nm for 5-c MeCbl (Δε_440_ = 4.8 mM^−1^cm^−1^) and 590 nm for ^ΔC^MTRR_sq_ (Δε_590_ = 2.8 mM^−1^cm^−1^). Using these values, a reasonable correspondence between the simulated and experimental difference spectra was obtained with 18 μM MeCbl (in a 2:1 ratio of 6-c:5-c) and 18 μM ^ΔC^MTRR_sq_ (Fig. 4*B*, *orange trace*). MeCbl (∼12 μM) was recovered by HPLC analysis of a reaction mixture that was set up in parallel with half the concentration of each component compared to the stopped-flow experiment (Fig. S1*C*). Biphasic kinetics were observed at 526 nm for 6-c MeCbl formation, with the majority of the amplitude change (∼90%) being associated with the slow phase, and an observed rate constant (*k*_*obs (total)*_) of 0.1 ± 0.01 s^−1^ (Fig. 4C, *black trace*). The monophasic kinetics of ^ΔC^MTRR_sq_ formation monitored at 590 nm, yielded a comparable *k*_*obs*_ of 0.1 ± 0.02 s^−1^ (Fig. 4*D*, *black trace*).

The difference spectrum obtained with P1173L MTR was strikingly different with major absorbance maxima at 464 nm and 590 nm (Fig. 4B, *blue spectrum*). The *k*_*obs (total)*_ for 6-c MeCbl (0.003 ± 0.002 s^−1^, 526 nm) and ^ΔC^MTRR_sq_ (0.009 ± 0.004 s^−1^, 590 nm) formation were ∼30- and 10-fold slower, respectively, than for wild-type MTR (Fig. 4, *C* and *D blue traces*). Due to the slower kinetics, data for P1173L MTR was collected over 200 s and the *k*_*obs*_ values were obtained from the exponential fits (Fig. S1*D*). The data revealed that the P1173L mutation slows down electron transfer from ^ΔC^MTRR_hq_ by at least an order of magnitude. Furthermore, the increase in absorbance at 464 relative to 526 nm observed with P1173L MTR (Fig. 4*B*), suggested that the mutation decreases the rate of 6-c MeCbl formation.

### Complex formation between MTR and ^ΔC^MTRR is not impaired by the P1173L mutation

We assessed whether the P1173L mutation impairs complex formation (*k*_*1obs*_) with ^ΔC^MTRR_hq_ that can be monitored by the loss of His-785 coordination, which is accompanied by an increase in absorbance at 464 nm (Fig. 4, *A* and *E*, i→ii). Biphasic kinetics were observed when either wild-type or P1173L MTR was mixed with ^ΔC^MTRR_hq_ with the slow phase accounting for ∼70 to 75% of the amplitude change. With both enzymes, the observed rate constant for complex formation was 0.1 ± 0.01 s^−1^ (Fig. 4*E*, *inset*). With wild-type MTR, the *k*_*obs*_ for complex formation, *i.e.*, conversion of (i) to (ii) is comparable to that for the formation of 6-c MeCbl, *i.e.*, conversion of (i) to (v), consistent with the first step being rate-limiting in the overall reactivation reaction (Fig. 4*A*). In contrast, the *k*_*obs*_ for complex formation with P1173L MTR is ∼30-fold faster than for the formation of 6-c MeCbl, indicating that the rate-limiting step in the reductive methylation reaction is altered by the mutation.

### EPR analysis of MTR-bound cob(II)alamin

**Figure 5:**
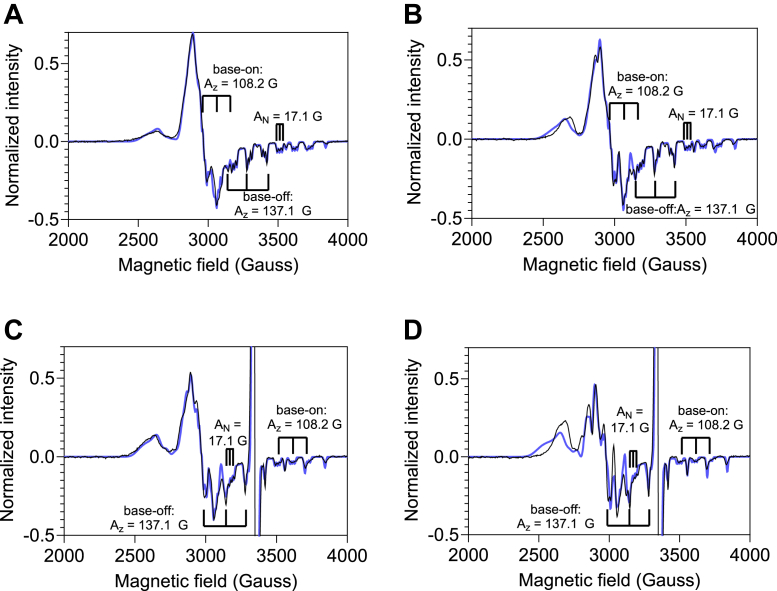
**EPR spectra of cob(II)alamin bound to wild-type or P1173L MTR.***A–B,* EPR spectra of cob(II)alamin (130 μM) bound to wild-type (*A*) or P1173L *(B)* MTR. The spectra are a mixture of base-off/His-on and base-off cob(II)alamin. *(C–D)* EPR spectra of cob(II)alamin (130 μM) bound to wild-type (*C*) or P1173L *(D)* MTR and mixed with ^ΔC^MTRR (200 μM) in 100 mM potassium phosphate buffer, pH 7.4, containing 10% glycerol. The spectra are a mixture of 5-c cob(II)alamin with nitrogen or oxygen ligands, albeit in slightly different ratios. The hyperfine (A_z_) and superhyperfine (A_N_) interactions are labeled. The sharp *g* = 2 signal corresponds to the ^ΔC^MTRR semiquinone. In each panel, the experimental (*black*) and simulated (*blue*) spectra obtained with the parameters listed in Table S1, are overlaid. EPR spectra were recorded at 80 K using the following parameters: 9.38-GHz microwave frequency, power 2 mW, modulation amplitude 10 G, modulation frequency 100 kHz, 3000 G sweep width centered at 3500 G, conversion time 164 ms, time constant 82 ms. 10 scans were collected per sample.

In *E. coli* MTR, the histidine ligand to cob(II)alamin is replaced by a water molecule in the interprotein complex with its physiological reducing partner, flavodoxin (41). We used electron paramagnetic resonance (EPR) spectroscopy to test whether MTRR similarly induces a histidine-to-water shift in the lower axial coordination position and if this equilibrium is perturbed by the P1173L mutation in MTR. Interactions between the unpaired electron in cob(II)almin and the ^59^Co nucleus (I = ^7^/_2_) result in hyperfine splitting into an 8-line spectrum while ^14^N coordination (I = 1) from His-785 leads to further superhyperfine splitting of each line into triplets, which are most clearly visible in the high-field region (2800–4000 G) of the spectrum (Fig. 5). Simulation of the EPR spectrum of wild-type MTR revealed that cob(II)alamin exists as a mixture with ∼71% nitrogen (A_x_ = 6.1 G, A_y_ = 5.7 G, A_z_ = 108.2 G, and A_N_ = 17.1 G) and ∼29% oxygen (A_x_ = 81.4 G, A_y_ = 72.5 G and A_z_ = 137.1 G) coordination (Fig. 5*A*). By comparison, cob(II)alamin bound to P1173L MTR was a mixture with ∼59% nitrogen and ∼41% oxygen coordination (Fig. 5*B*).

In the presence of ^ΔC^MTRR, the proportion of oxygen-coordinated cob(II)alamin increased in both wild-type (from 29 to 49%) and P1173L MTR (41–62%) (Fig. 5, *C* and *D*). The additional *g* = 2.0 signal observed in the presence of ^ΔC^MTRR, was assigned to the stable FMN semiquinone, which is a minor component of the purified protein. The EPR data reveal that despite minor differences in the relative distribution of 5-c cob(II)alamin with His-785 *versus* H_2_O ligand, complex formation with ^ΔC^MTRR shifts the equilibrium toward H_2_O ligation in both wild-type and P1173L MTR.

### P1173L MTR is impaired in the formation of 6-c MeCbl during reductive methylation

**Figure 6:**
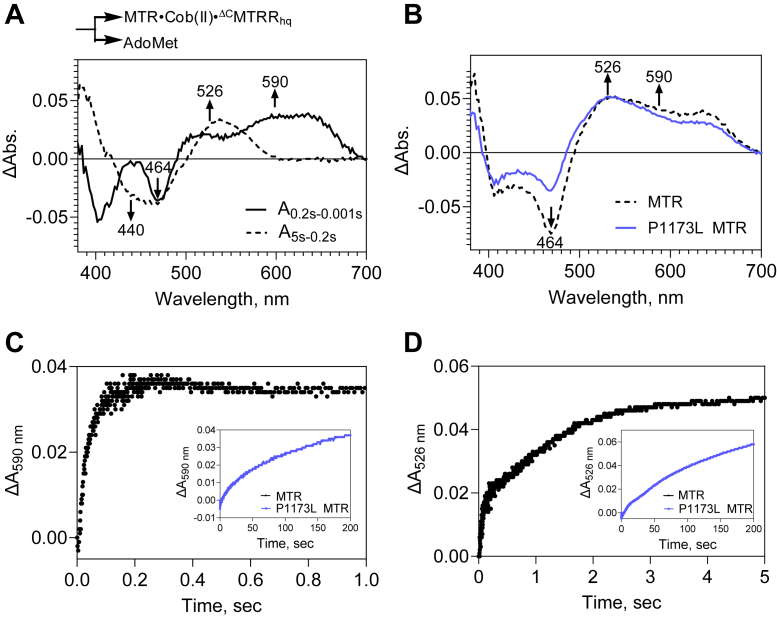
**P1173L MTR is impaired at multiple steps following the formation of the interprotein complex formation with ^^Δ^C^MTRR.***A,* Cob(II)alamin-bound MTR (40 μM) was pre-incubated with ^ΔC^MTRR_hq_ (70 μM) and rapidly mixed with AdoMet (400 μM) in an anaerobic stopped-flow spectrophotometer and spectra were monitored over 5 s. The difference spectrum associated with the fast phase (*solid trace*) shows an increase at 590 nm, indicating ^ΔC^MTRR_sq_ formation while the increase at 526 nm in the slow phase (*dotted trace*) indicates 6-c MeCbl formation. *B,* the same experiment as (*A*) but with P1173L MTR revealed that the majority (∼95%) of the reaction occurred in a single slow phase, which resulted in an increase in absorbance at 526 nm and 590 nm, indicating the formation of 6-c MeCbl and ^ΔC^MTRR_sq_ respectively. The difference spectrum for P1173L MTR is similar to that obtained with wild-type MTR (*dotted black trace*) after 5 s (*i.e.*, 5 s −0.001 s). *C,* time-dependent change in absorbance at 590 nm seen with wild-type (*black*) and P1173L MTR (*inset, blue*) yielded rate constants of 27.5 ± 3 s^−1^ and 0.007 ± 0.001 s^−1^, respectively. *D,* time-dependent change in absorbance at 526 nm with wild-type MTR (*black trace*) is biphasic with observed rate constants of 20.5 ± 2.9 s^−1^ and 0.7 ± 0.03 s^−1^, respectively. The majority of the amplitude change at 526 nm for P1173L MTR (*inset, blue trace*) is associated with the slow phase (0.007 ± 0.0002 s^−1^). The data are representative of three independent experiments.

We assessed whether the steps following complex formation between MTR and ^ΔC^MTRR are impaired by the P1173L mutation. For this, we pre-mixed MTR•cob(II)alamin with ^ΔC^MTRR_hq_ to form the interprotein complex, which was then rapidly mixed with AdoMet (Fig. 6*A*). With wild-type MTR, the difference spectra could be separated into two distinct phases. In the first, extending from 1 msec to 0.2 s, an increase in absorbance at 590 nm was seen, indicating the formation of ^ΔC^MTRR_sq_ and a corresponding decrease at 464 nm, indicating reductive methylation of cob(II)alamin (Fig. 6*A*, *solid trace*). We assign this phase to electron transfer from ^ΔC^MTRR_hq_ to form cob(I)alamin and subsequent methylation to form 5-c MeCbl (Fig. 4*A*, ii→iv).

In the second phase extending from 0.2 to 5 s, an increase in absorbance at 526 nm and a decrease at 440 and 464 nm was observed (Fig. 6*A*, *dotted trace*). Since no change is observed at 590 nm, we assign this phase to the formation of 6-c from 5c-MeCbl (Fig. 4*A*, iv→v). With P1173L MTR, the reaction required 200 s for completion and the difference spectrum resembled the combination of the two phases seen with wild-type MTR (Fig. 6*B*). We assign the difference spectrum to the conversion of cob(II)alamin in the P1173L MTR- ^ΔC^MTRR_hq_ complex to 6c-MeCbl (Fig. 4*A*, ii→v). The kinetics of semiquinone formation at 590 nm in the fast phase (Fig. 6*C*, *black trace*) was seen only with wild-type MTR and represents a complex rate constant (27.5 ± 3 s^−1^) that includes electron transfer to cob(I)alamin as well as its subsequent methylation to MeCbl. The second slower phase exhibited negligible absorbance change at 590 nm, indicating that once formed, the ^ΔC^MTRR_sq_ was stable under our experimental conditions. The biphasic kinetics at 526 nm yielded a rate constant for the fast phase (20.5 ± 2.9 s^−1^) that was similar to that obtained for semiquinone formation at 590 nm, while the slower phase (0.7 ± 0.03 s^−1^) corresponded to the conversion of 5-c to 6-c MeCbl (Fig. 6*D*, *black trace*). With P1173L MTR, the formation of 6-c MeCbl from the preformed interprotein complex took place such that most (∼95%) of the amplitude change at 590 nm (Fig. 6C, *inset*) and 526 nm (Fig. 6*D*, *inset*) was associated with the slow phase with rate constants of 0.007 ± 0.001 s^−1^ and 0.007 ± 0.0002 s^−1^, respectively. These data revealed that the P1173L mutation decreases the rate of 6-c MeCbl formation 100-fold but also pointed to an additional impairment in the rate of electron transfer to cob(II)alamin.

### P1173L MTR is impaired in cob(I)alamin methylation and entry into catalysis

**Figure 7:**
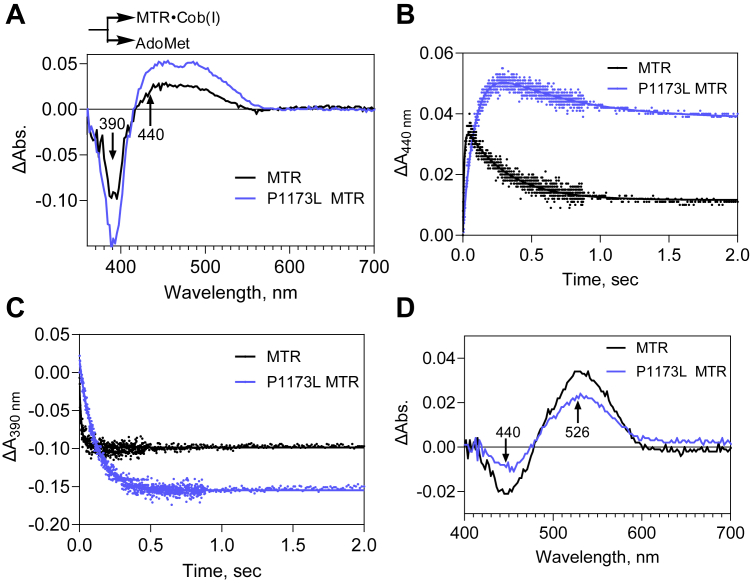
**Kinetics of cob(I)lamin methylation and conformational change for entry into catalysis is impaired in P1173L MTR:***A,* Cob(II)alamin (20 μM) bound to wild-type or P1173L MTR was reduced to cob(I)alamin with titanium (III) citrate, and then rapidly mixed with AdoMet (200 μM) Difference spectra observed during the first phase with wild-type (0.001–0.05 s, *black*) and P1173L (0.001–0.3 s, *blue*) MTR shown an increase at 440 nm and a decrease at 390 nm. *B,* time-dependent increase in absorbance in the first phase at 440 nm with wild-type (*black*) and P1173L (*blue*) MTR yielded rate constants for cob(I)alamin methylation of 110.3 ± 11.8 s^−1^ and 12.7 ± 0.8 s^−1^, respectively. The decrease in 440 nm absorbance in the second phase of the reaction corresponds to the 5-c to 6-c MeCbl conversion at 1.4 ± 0.1 s^−1^ for P1173L MTR and 2.8 ± 0.5 s^−1^ wild-type MTR. *C,* Cob(I)alamin methylation was also monitored by the decrease in absorbance at 390 nm and yielded rate constants of 86.4 ± 21.4 s^−1^ for wild-type MTR (*black*) and 8.4 ± 0.3 s^−1^ for P1173L MTR (*blue*). *D,* spectral changes in the second phase of the reaction*,* which was monitored up to 2 s*,* show that the decrease at 440 nm is accompanied by an increase at 526 nm in wild-type (*black*) and P1173L (*blue*) MTR. The data are representative of three independent experiments.

The kinetics of cob(I)alamin methylation (*k*_*3obs*_) and conversion of 5-c to 6-c MeCbl (*k*_*4obs*_) were monitored by reducing MTR•cob(II)alamin with titanium(III)citrate to generate cob(I)alamin, which was then rapidly mixed with AdoMet under anaerobic conditions (Fig. 7*A*). Two distinct phases were discernable in the difference spectra for both wild-type and P1173L MTR. In the first phase, the decrease at 390 nm (cob(I)alamin) was accompanied by an increase at 440 nm (5-c MeCbl) and lasted between 0.001 to 0.05 s with wild-type MTR (Fig. 7*A*, *black*). Similar *k*_*obs*_ values were obtained from the rate of change at 440 nm (110.3 ± 11.8 s^−1^) and 390 nm (86.4 ± 21.4 s^−1^) (Fig. 7, *B* and *C*, *black*). For P1173L MTR, the first phase lasted between 0.001 to 0.3 s and yielded rate constants of 12.7 ± 0.8 s^−1^ (monitored at 440 nm) and 8.4 ± 0.3 s^−1^ (at 390 nm) (Fig. 7, *A* and *B*, *blue*), which are ∼8 to 10 fold slower than for wild-type MTR. In the second phase (Fig. 7*D*), the decrease at 440 nm (5-c MeCbl) was accompanied by an increase at 526 nm (6-c MeCbl) and lasted between 0.05 to 2 s for wild-type and 0.3 to 2 s for P1173L MTR. With wild-type MTR, a *k*_*obs*_ of 2.8 ± 0.5 s^−1^ (Fig. 7*B*, *black*) was estimated from the kinetics at 440 nm, which was 2-fold higher than for P1173L MTR (1.4 ± 0.1 s^−1^) respectively.

## Discussion

**Figure 8:**
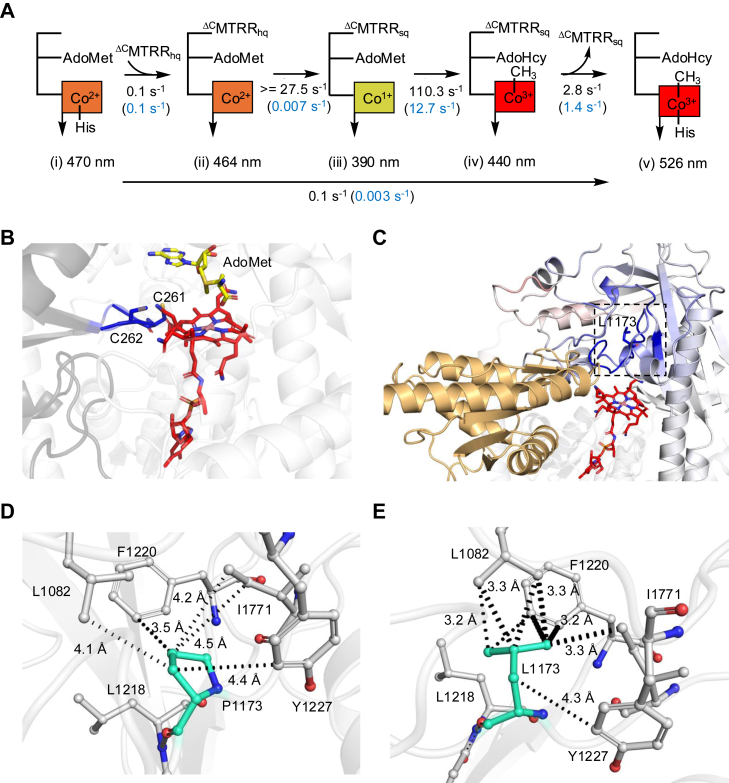
**The electron transfer step is most significantly impacted in P1173L MTR.***A,* Comparison of the rate constants for wild-type (*black*) *versus* P1173L (*blue*) MTR repair reaction. *B,* AlphaFold2 model of the complex between ^ΔN^MMADHC (*dark gray*) and ^ΔN^MTR (*light gray*) predicts that the beta-hairpin (*blue*) containing Cys-261 and Cys-262 is docked close to B_12_ (*red*); AdoMet (*yellow*) is proximal to B_12_. *C,* protein flexibility analysis of P1173L MTR aligned with the AlphaFold2 predicted ^ΔC^MTRR-MTR complex. MTR and ^ΔC^MTRR are shown in *light gray* and *sand* respectively. The regions of MTR that are predicted to be less flexible due to the P1173L mutation are denoted by a gradient from *lighter* to *darker blue*, indicating a decrease in flexibility. The *dotted black box* is magnified in (*D*) and (*E*)*. D–E,* Protein flexibility analysis of wild-type (*D*) and P1173L (*E*) MTR shows that Pro-1173 (*cyan sticks*) is engaged in weak hydrophobic interactions (>4 Å) (*dotted black lines*) with its neighboring residues with the wild-type protein but that these interactions are stronger in the variant (3.2–3.3 Å). More hydrophobic interactions (*dotted black lines*) between Leu-1173 and its neighboring residues are seen in the variant.

Disease-causing mutations are Nature’s map of residues that are essential for protein function. While characterization of clinical variants in the mitochondrial branch of the B_12_ trafficking pathway has provided rich insights into protein structure and function (6, 7, 8, 9, 10), studies on patient mutations in the cytoplasmic branch have been limited (42). As with other autosomal recessive diseases, mutations in MTR are typically found in a compound heterozygous state (33), which often limits the direct correlation between genotype and disease phenotype, *e.g.*, severity, age of onset, and B_12_ responsiveness. Hence, characterization of the biochemical penalties associated with individual patient mutations can be potentially informative for therapeutic options. In this study, we report the kinetic characterization of full-length human MTR and reveal that P1173L, the most common clinical variant, impairs the repair cycle (Fig. 8*A*).

While multiple approaches are commonly used to solve the problem of recombinant expression for soluble proteins that are prone to aggregation, full-length MTR has been unusually recalcitrant to efforts that were initiated almost 2 decades ago (36). In addition to the common strategies for increasing soluble expression like chemical and cold chaperones, codon optimization, use of plasmids with N- or C-terminal tags or fusions, co-expression with MTRR, which reportedly stabilized human MTR (36), failed to improve yield in our hands. The challenge with expressing human MTR was surprising since recombinant *E. coli* MTR, which shares high sequence similarity, can be purified in a relatively high yield (43). However, *E. coli* lacks homologs of the MMACHC and MMADHC chaperones, and its divergence from human MTR is indicated by the fact that their respective redox partners are not interchangeable, *i.e.* MTRR for human MTR and the flavodoxin/flavodoxin reductase system for *E. coli* MTR (36). We found that co-expression of ^ΔN^MMADHC, representing the well-folded core of this chaperone, was key to stabilizing recombinant apo-human MTR, highlighting its potential importance for regulating MTR stability and hence its activity, in mammalian cells (Fig. 2*A*).

Limited characterization of mammalian MTR purified from human placenta (44) and pig liver (45) has been reported previously. The specific activity of recombinant human MTR determined in this study is comparable to that for the native pig liver enzyme (1.6 μmol min^−1^ mg^−1^ at 37 °C in the DTT/B_12_ assay) and ∼100-fold higher than that reported for the placental enzyme. Further, while the *K*_*M*_ for homocysteine is comparable (3 ± 2 μM *versus* 2.1 ± 1.6μM) the *K*_*M*_ for *6S*-CH_3_-H_4_F is considerably higher for recombinant human (108 ± 18 μM) *versus* native porcine liver MTR (12 ± 2 μM). The reason for this difference is not understood.

A recent study from our laboratory reported the mechanism of B_12_ loading from MMADHC onto a truncated MTR variant, comprising only the B_12_ and AdoMet-binding domains (17) (Fig. 3*A*). The loading mechanism exploits the chemical lability of the Co-S bond and uses the vicinal Cys-262 thiolate on MMADHC to initiate heterolytic cleavage. In this study, we confirmed that AdoMet serves as the methyl group donor following B_12_ loading, but also found that CH_3_-H_4_F, and to a lesser extent H_4_F, promote the repair reaction (Fig. 3, *B* and *C*). We used AlphaFold2 (37) to model the interaction between ^ΔN^MMADHC and ^ΔN^MTR. In the model, the β-hairpin element in MMADHC on which Cys-261 and Cys-262 reside, is seen to be reaching into the B_12_ domain of MTR, with AdoMet positioned in close proximity (Fig. 8*B*). A combined cryoEM and AlphaFold2 analysis of a bacterial MTR revealed that CH_3_-H_4_F binding promotes uncapping of the B_12_ domain, making it available for interactions with other domains (28). We postulate that the presence of CH_3_-H_4_F during B_12_ loading from MMADHC favors the reactivation conformation exposing the B_12_ domain for interaction with the AdoMet domain.

A detailed steady-state and pre-steady state kinetic analysis helped isolate the biochemical penalty in P1173L MTR to the repair rather than the catalytic cycle and identified multiple steps that are impacted by the mutation. The impairment in the repair cycle was masked in the presence of the artificial DTT/B_12_ reducing system run under *V*_*max*_ conditions, although the mutation increases the *K*_M_ for AdoMet ∼40 fold to 8 ± 2 μM (Fig. 2*E*, Table 1). Assuming that the *K*_M_ corresponds to *K*_D_ to a first approximation, the AlphaFold2 model of human MTR provides a framework for interpreting the higher *K*_M_ value. The model predicts that the P1173L mutation disrupts interactions between AdoMet and two highly conserved flanking residues (Arg-1172 side chain and Ala-1174 backbone) (Fig. 3*D*). The *K*_M_ for AdoMet for P1173L MTR (8 ± 2 μM) predicts that its activity would be more susceptible in some tissues than in others; tissue AdoMet concentrations are reported to vary from <4 to 60 μM (46). However, the major penalty incurred by the P1173L mutation appears to be its ∼30-fold lower activity in the presence of the MTRR/NADPH reducing system (Table 1), and impeded reentry from the repair to the catalytic cycle.

Pre-steady state kinetic and EPR analyses revealed that complex formation with ^ΔC^MTRR_hq_, which represents the slow step (*k*_*obs*_ = 0.1 s^−1^) in the repair cycle for wild-type MTR, is unaffected by the P1173L mutation (Fig. 8A). Reduction of cob(II)alamin to cob(I)alamin on MTR is energetically unfavorable and kinetically coupled to a highly exergonic methylation reaction (40). The rate constant for cob(I)alamin formation is challenging to measure directly. With wild-type MTR, the complex rate constant for electron transfer to cob(II)alamin and subsequent methylation to form 5-c MeCbl is 27.5 ± 3 s^−1^, while the *k*_*obs*_ for cob(I)alamin methylation alone is 110.3 ± 11.8 s^−1^. From these data, we infer that the rate constant for the electron transfer step is at least 27.5 s^−1^ for wild-type MTR (Fig. 8*A*).

With P1173L MTR, the overall reductive methylation reaction proceeds with a *k*_*obs*_ = 0.003 ± 0.002 s^−1^ (Fig. 4*C*), which is considerably slower than the rate constants for the individual steps in the repair cycle catalyzed by P1173L MTR. The rate constant associated with the electron transfer step could not be experimentally separated from the subsequent methylation step for P1173L MTR and is assigned as the rate-limiting step based on the following analysis. When the pre-formed P1173L MTR•cob(II)alamin•^ΔC^MTRR_hq_ complex was rapidly mixed with AdoMet, the rate constants for ^ΔC^MTRR_sq_ and 6-c MeCbl formation were identical (0.007 ± 0.001 s^−1^). The rate constant for cob(I)alamin methylation was 12.7 ± 0.8 s^−1^ and for the conversion of 5-c to 6-c MeCbl was 1.4 ± 0.1 s^−1^. We, therefore, conclude that electron transfer from MTRR to cob(II)alamin represents the slow step with a rate constant of ∼0.007 s^−1^, which given the low value, is likely to be the same as the overall *k*_*obs*_ for the repair reaction, *i.e.*, 0.003 s^−1^ (Fig. 8*A*).

We performed protein flexibility analysis using DynaMut (47), a computational tool for predicting the impact of a mutation on protein conformation, flexibility, and stability. This analysis revealed that the P1173L mutation increases the folding free energy (ΔΔG) by +1.927 kcal mol^−1^ and decreases the vibrational entropy energy by 0.644 kcal mol^−1^ K^−1^ relative to the wild-type protein. Combined, these changes predict that the mutation increases overall protein stability but decreases flexibility. The secondary structures surrounding the mutated residue are predicted to become more rigid and happen to reside on the same face that is predicted to engage with MTRR (Fig. 8*C*). In wild-type MTR, Pro-1173 is expected to engage in weak hydrophobic interactions with the neighboring Leu-1082, Ile-1771, Leu-1218, Phe-1220, and Tyr-1227, with most of the interactions being at a >4 Å distance (Fig. 8*D*). However, in P1173L MTR, the same interactions with Leu-1173 are stronger with distances ranging from 3.2 to 3.3 Å, and additional hydrophobic interactions are predicted (Fig. 8*E*). Furthermore, the backbone of Leu-1173 is predicted to engage in additional polar interactions with the backbone of Leu-1218. Notably, the protein face that is predicted to become more rigid due to the P1173L mutation also interacts with MTRR for the electron transfer step preceding methylation. Based on our analysis, we postulate that stronger interactions between Leu-1173 and adjacent residues decrease MTR flexibility and impede electron transfer, which has been seen in other protein complexes such as in photosystem II (48).

In summary, we have stabilized and been able to purify recombinant full-length human MTR and the clinical P1173L variant, which is the most common one found in MTR-deficient homocystinuria patients. Detailed kinetic characterization has identified a change in the rate-limiting step, *i.e.* from the complex formation with MTRR in wild type, to electron transfer from MTRR in P1173L MTR, leading to an overall 30-fold lower rate of repair. Clinically, patients with MTR mutations typically receive B_12_, betaine and folic acid supplementation therapy (49, 50). Our data predict that the efficacy of B_12_ supplementation in patients carrying the P1173L mutation might be enhanced in combination with an electron donor like DHLA as used in our *in vitro* assay, to help bypass the physiological repair system.

## Experimental procedures

### Reagents

Cob(II)alamin was generated by photolysis of adenosylcobalamin as previously described (51). OH_2_Cbl (Cat # 95200) and MeCbl (Cat #M9756) were purchased from Sigma-Aldrich. AdoMet was from Cayman Chemicals, MI (Cat # 13956). Isopropyl β-D−1−thiogalactopyranoside (IPTG) (catalog no. I2481C) was from Gold Biotechnology. (*6S*)-CH_3_-H_4_F was from Matrix scientific. D,L-Homocysteine was from Sigma-Aldrich (Cat #H4628). Protocatechuate was from Cayman Chemicals, MI (Cat # 14916) and protocatechuate 3,4 dioxygenase was from Fisher Scientific (Cat # ICN15197505). Ni(II)-NTA resin (Cat # 30210) was from Qiagen, and glutathione sepharose resin (Cat # 17513201) was from Cytiva. Primers were purchased from Integrated DNA Technologies.

### Cloning of MTR and P1173L MTR

The cDNA for human MTR (synthetic gene obtained from Genscript) was codon optimized for expression in *E. coli* and cloned into a pET-28 vector between the *Bam*H1 and *Xho*1 restriction sites to generate a construct with two 6x His tags, one each at the N and C-termini, respectively, and with a thrombin cleavage site at the N-terminus to generate, pET28-thrombin-MTR. Pro-1173 was mutated to a leucine using the wild-type expression construct and the following primers (the mutant codon is underlined).

Forward primer: 5′-AAA GGT ATT CGT CTG GCA CCG GGT-3′

Reverse primer was 5′-ACC CGG TGC CAG ACG AAT ACC TTT-3′.

### Co-expression of recombinant MTR with ^ΔN^MMADHC

*E. coli* BL21 (DE3) cells were transformed with pET28-thrombin-MTR and pGEX- ^ΔN^MMADHC (16) constructs with kanamycin and ampicillin resistance genes, respectively and were grown overnight at 37 °C in 100 ml Luria−Bertani (LB) medium containing kanamycin (50 *μ*g/ml) and ampicillin (100 *μ*g/ml). Then, 6 × 1 L LB medium containing kanamycin (50 *μ*g/ml) and ampicillin (100 *μ*g/ml) was inoculated with 12 ml of the starter culture and grown at 37 °C. After ∼3 h, when the OD_600_ had reached ∼0.6 to 0.8, the temperature was lowered to 15 °C. Expression was induced with 50 *μ*M IPTG, and cells were harvested 18 − 20 h later. Cell pellets were stored at −80 °C until use. The same protocol was adopted for co-expression of P1173L MTR with ^ΔN^MMADHC.

### Purification of MTR

Cell pellets were suspended in 200 ml Buffer A (50 mM Tris−HCl, pH 8.0, 500 mM KCl, 15 mM imidazole and 10% glycerol) supplemented with 10 mM *β*-mercaptoethanol (*β*-ME), and 0.15 mg/ml lysozyme, and one tablet of EDTA-free cOmplete Protease Inhibitor Cocktail (Roche), and the cell suspension was stirred at 4 °C for 30 min and sonicated (power setting = 5) on ice for 14 min at 15 s intervals separated by 45 s cooling periods. The sonicate was centrifuged at 38,000*g* for 45 min, and the supernatant was loaded onto a Ni-NTA agarose column (2.5 × 5 cm, Qiagen) pre-equilibrated with Buffer A. The column was washed with 200 ml of Buffer A supplemented with 45 mM imidazole and eluted with 100 ml of Buffer A, supplemented with 400 mM imidazole. Fractions containing MTR were identified by SDS-PAGE analysis, pooled, buffer exchanged into Buffer B (50 mM Tris, pH 8, 500 mM KCl, and 10% glycerol) and concentrated in Amicon centrifugation devices (MWCO = 50 kDa, Sigma-Aldrich). The protein monomers were separated from oligomers by size exclusion chromatography on a Superdex 200 column (120 ml, GE Healthcare) equilibrated with Buffer B. Fractions corresponding to the MTR monomer were concentrated, pooled, and stored at −80 °C until use. The same protocol was followed for purifying P1173L MTR. Approximately 3 mg each of wild-type and P1173L MTR were obtained per liter of culture.

### Cloning, expression, and purification of ^ΔC^MTRR

A previously reported construct of full-length human MTRR in the pGEX vector with an N-terminal GST tag was used to clone ^ΔC^MTRR, which contained residues from 1 to 231, spanning the FMN domain (23) using the following primers.

Forward primer 5′-TCA CTT ACC CGT TAG GTA CCC CCA CTC-3′

Reverse primer: 5′ GAG TGG GGG TAC CTA ACG GGT AAG TGA 3′

The ^ΔC^MTRR protein was expressed as a GST fusion protein by transforming in *E. coli* strain BL21(DE3), using a modified version of the protocol described previously (23). Briefly, an overnight culture of *E. coli* containing the expression construct was grown at 37 °C in an LB medium containing ampicillin (100 *μ*g/ml). Then, six x 1 L modified Terrific Broth (20 g yeast extract, 10 g bactotryptone, 4 ml of glycerol, 4.33 g of Na_2_HPO_4_, and 2.65 g of KH_2_PO4), with 100 *μ*g/ml ampicillin, was inoculated with 12 ml of overnight culture and grown in a shaker at 28 °C. When the OD_600_ reached ∼1.0 after 5 h, IPTG was added to a final concentration of 100 μM, and the temperature was lowered to 25 °C. The cells were grown overnight, pelleted by centrifugation at 5000*g* for 30 min, and stored at −80 °C until further use. The frozen cells were thawed and resuspended in 200 ml of GST wash/bind buffer (4.3 mM Na_2_HPO_4_, 1.47 mM KH_2_PO_4_, 137 mM NaCl, 2.7 mM KCl, 0.5 mM EDTA, and 1 mM DTT) supplemented with lysozyme (200 *μ*g/ml) and one tablet of EDTA-free cOmplete Protease Inhibitor Cocktail (Roche) and stirred at 4 °C for 30 min. Cells were sonicated (power setting = 5) on ice for 8 min at 15 s intervals separated by 60 s cooling periods. The sonicate was centrifuged at 38,000*g* for 45 min to pellet cell debris and insoluble matter. The resulting supernatant was loaded onto a Glutathione Sepharose 4B column, previously equilibrated with GST wash/bind buffer. Nonspecifically bound proteins were washed from the column with 150 ml GST wash/bind buffer, and GST-fused ^ΔC^MTRR was eluted with 10 mM GSH in elution buffer (50 mM Tris-HCl, pH-7.6, 0.5 mM EDTA, and 1 mM DTT). The GST tag was removed by limited proteolysis with thrombin (Baxter International Inc.). The protein was exchanged into 50 mM potassium phosphate buffer, pH 7.0 by overnight dialysis at 4 °C. Cleaved ^ΔC^MTRR was reapplied to the Glutathione-Sepharose 4B column equilibrated with GST wash/bind buffer. ^ΔC^MTRR was separated from the remaining glutathione-binding proteins by washing the column with GST wash/bind buffer and concentrated in Amicon centrifugation devices (MWCO = 10 kDa, Sigma-Aldrich). The protein monomers were separated from oligomers by size exclusion chromatography on a Superdex 200 column (120 ml, GE Healthcare) equilibrated with a buffer containing 50 mM potassium phosphate, pH 7.0, and 10% glycerol. Fractions containing the ^ΔC^MTRR monomers were pooled and concentrated using Amicon centrifugation devices (MWCO = 10 kDa, Sigma-Aldrich). The concentrated protein was incubated with equimolar FMN at room temperature for 5 min before passing through a NanoSep filter column (MWCO = 10 KDa, PALL) to get rid of excess unbound FMN. The concentration of purified recombinant ^ΔC^MTRR was determined spectroscopically, using a molar extinction coefficient of 14,700 M^−1^cm^−1^ at 454 nm (25). Approximately 4 mg of ^ΔC^MTRR was obtained per liter of culture. The same protocol was used to purify full-length MTRR, which was obtained in a yield of ∼2 mg per liter of culture.

### Expression and purification of ^ΔN^MMADHC

MMADHC lacking the first 115 residues (^ΔN^MMADHC) was purified as described previously using the pGEX-^ΔN^MMADHC construct (16).

### Formation of B_12_-bound ^ΔN^MMADHC

B_12_-bound ^ΔN^MMADHC was prepared as described previously (16) by mixing OH_2_-Cbl (100 *μ*M) with equimolar ^ΔN^MMADHC in Buffer C (100 mM HEPES, pH 7.4, 150 mM KCl and 10% glycerol) at room temperature for 1 h. Unreacted OH_2_-Cbl was removed by centrifugation using a NanoSep filter column (MWCO = 10 kDa, PALL).

### DTT/OH_2_Cbl-dependent MTR assay

MTR activity was measured using non-radioactive assays as described previously (52). The reaction mixture (800 μl total volume) containing 50 mM DTT, 10 μM H_2_OCbl, 300 μM D, L-homocysteine, 100 μM AdoMet and 50 nM MTR/P1173L MTR (pre-incubated with five-fold excess of MeCbl) in Buffer D (100 mM potassium phosphate buffer, pH 7.4) was incubated at 37 °C for 5 min following, which 250 μM (*6S*)-CH_3_-H_4_F was added to initiate the reaction. 150 μl aliquots of the reaction mixture were removed at the desired time points and quenched by adding 37.5 μl of a 1:1 mixture of formic acid: HCl. The reaction was heated at 80 °C for 10 min. Once cool, the concentration of the methenyl-H_4_F product formed was determined using an extinction coefficient of 26.5 × 10^3^ M^−1^ cm^−1^ at 350 nm. The assays were conducted at varying substrate concentrations to generate a Michelis-Menten plot for each substrate. For each reaction, the initial velocity (v) was obtained from the slope of the line fitted to Equation 1:

(1) $$\left[P\right]=v*t$$

where [P] is the concentration of product formed, and t is the time of the reaction.

For the Michelis-Menten analysis, the dependence of initial velocities on substrate concentration was plotted and fitted to Equation 2:

(2) $$V=\frac{V_{\max}\;*\;\left[S\right]}{K_{M}+\left[S\right]}$$

where [S] is the substrate concentration, V_max_ is the maximal velocity and *K*_M_ is the substrate concentration at half V_max_. The *K*_*M*_ value for homocysteine is reported for the L-isomer.

### MTRR/NADPH-dependent MTR assay

MTR activity was measured using the non-radioactive assays as described previously (52). The reaction mixture (800 μl total volume) containing 300 μM D, L-homocysteine, 100 μM AdoMet, 1 μM MTRR and 50 nM MTR/P1173L MTR (pre-incubated with a 5-fold excess of MeCbl) in 100 mM potassium phosphate buffer, pH 7.4 was incubated at 37 °C for 5 min following which 250 μM CH_3_-H_4_F was added and incubated at 37 °C for an additional 2 min. The reaction was initiated by the addition of 60 μM NADPH. Aliquots (150 μl) of the reaction mixture were removed at the desired times (typically 2, 4, 6, 8, and 10 min) and quenched by adding 37.5 μl of a 1:1 mixture of formic acid:HCl. The reaction was heated at 80 °C for 10 min and cooled, which led to methenyl-H_4_F formation. The concentration of methenyl-H_4_F was determined at 350 nm using an extinction coefficient of 26.5 × 10^3^ M^−1^ cm^−1^. MTR activity was determined at varying MTRR concentrations. For each reaction, the initial velocity was obtained from the slope using equation 1 above.

The dependence of initial velocity on MTRR concentration was fitted to the Hill Equation (3):

(3) $$V=\frac{\left(V_{\max}\;*\;{\left[S\right]}^{n}\right)}{K{\text{act}}^{n}+{\left[S\right]}^{n}}$$

where [S] is the MTRR concentration, V_max_ is the maximum velocity and *K*_act_ is the MTRR concentration at half maximal V_max_ and n is the Hill coefficient.

### HPLC analysis of MeCbl formed during cofactor loading onto MTR

Buffer C was purged with N_2_ gas in a sealed anaerobic bottle for 45 min and cycled into an anaerobic chamber where it was stored overnight. B_12_ bound to ^ΔN^MMADHC was prepared aerobically as described above and exchanged into anaerobic Buffer C in the anaerobic chamber (<0.5 ppm of O_2_) using a NanoSep filter device (MWCO = 10 kDa, PALL). A 100 μl stock solution of MTR was purged with N_2_ gas on ice for 45 min in a sealed 1.5 ml Eppendorf tube before being moved into the anaerobic chamber. To determine the methyl group donor following B_12_ transfer to MTR, 250 μM each of AdoMet or CH_3_-H_4_F or both were mixed with 50 μM MTR at room temperature for 5 min in the anaerobic chamber, following which B_12_-bound ^ΔN^MMADHC (20 μM) was added. The reaction was quenched after 60 min by the addition of 2% trifluoroacetic acid (TFA) and centrifuged at 13,600*g* for 10 min. The supernatant was used to detect the cobalamin species by HPLC analysis.

Samples (100 μl) were injected into an Alltima HP C18 column (Grace, 250 mm × 4.6 mm, 5 μM) at a flow rate of 1 ml/min. The mobile phase consisted of solvent A: 0.1% TFA in water and solvent B: 0.1% TFA in acetonitrile. Samples were eluted at a flow rate of 1 ml/min with a gradient from eight to 32% solvent B for 35 min, and the absorbance was monitored at 460 nm. The MeCbl (25 μM) and OH_2_Cbl (25 μM) standards were separated under the same conditions.

### Native gel analysis

The interprotein complex between MTR and MTRR was formed by mixing 15 μM cob(II)alamin-bound wild-type or P1173L MTR with an equimolar concentration of MTRR in Buffer C (15 μl) and incubated at room temperature for 5 min. For catalytic reactions, samples containing wild-type or P1173L MTR were mixed with equimolar concentration of MTRR and saturating concentrations of D, L-homocysteine, CH_3_-H_4_F, AdoMet and NADPH in a total volume of 15 μl. All samples were mixed with 15 μl 2X native gel-loading buffer (62.5 mM Tris−HCl, pH 6.8, 40% glycerol, 0.01% bromophenol blue).10 μl of each sample was loaded onto a 4–15% gradient gel (Bio-Rad) and separated using running buffer (2.5 mM Tris, 19.2 mM glycine, pH 8.3) at 4 °C, 100 V for 160 min.

### EPR spectroscopy

Samples were prepared in an anaerobic chamber as follows: For preparing MTR-bound cob(II)alamin, 200 μM wild-type or P1173L MTR was mixed with 130 μM cob(II)alamin in Buffer D containing 10% glycerol in a total volume of 250 μl. For preparing MTR-^ΔC^MTRR complex, 130 μM cob(II)alamin bound MTR/P1173L MTR was mixed with 200 μM ^ΔC^MTRR in Buffer D containing 10% glycerol in a total volume of 250 μl. The samples were incubated for 30 min at room temperature, transferred to EPR tubes, and frozen in liquid nitrogen.

EPR spectra were recorded on a Bruker EMX300 instrument equipped with a Bruker 4201 cavity and a ColdEdge cryostat (Miami University). The temperature was controlled with an Oxford Instruments MercuryiTC temperature controller. EPR spectra were recorded at 80 K using the following parameters: 9.37 GHz microwave frequency, power 2 mW, modulation amplitude 10 G, modulation frequency 100kHz, 3000 G sweep width centered at 3500 G, conversion time 164 msec,1024 data points, and time constant 82 msec. 10 scans were collected per measurement. EPR simulations were performed using the EasySpin program (53) using parameters described in Table S1.

### Photoreduction of ^ΔC^MTRR

For photoreduction of ^ΔC^MTRR, a 100 μl stock solution of ^ΔC^MTRR in 50 mM potassium phosphate, pH 7.0 containing 10% glycerol was purged with N_2_ gas on ice for 45 min in a sealed 1.5 ml Eppendorf tube before being moved into the anaerobic chamber. Inside the chamber, 140 μM ^ΔC^MTRR was mixed with 500 μM EDTA, 5 μM riboflavin, 500 μM protocatechuate, and 170 μg protocatechuate 3,4- dioxygenase in buffer D (800 μl) in a sealed cuvette and incubated at room temperature for 30 min. The sealed cuvette was placed in a beaker containing water at room temperature with constant stirring and was irradiated with a 600 W tungsten/halogen lamp through the side of the beaker. Irradiation was done for 10 s durations to avoid excessive sample heating and a spectrum was recorded after each irradiation. After ∼12 irradiations, a complete conversion to the hydroquinone (^ΔC^MTRR_hq_) was observed.

### Stopped-flow absorption spectroscopy

All concentrations noted for stopped-flow studies refer to final concentrations after mixing. For these studies, a 100 μl stock solution of wild-type or P1173L MTR in Buffer B was purged with N_2_ gas on ice for 45 min in a sealed 1.5 ml Eppendorf tube before being moved into the anaerobic chamber. Inside the chamber, 80 μM cob(II)alamin bound to wild-type or P1173L MTR was mixed with 500 μM protocatechuate and 170 μg protocatechuate 3,4- dioxygenase in Buffer D (450 μl) and incubated at room temperature for 30 min to get rid of any remaining oxygen.

To measure AdoMet-dependent reductive methylation, MTR•cob(II)alamin (40 μM) pre-incubated with AdoMet (400 μM) in Buffer D was rapidly mixed with ^ΔC^MTRR_hq_ (70 μM) in the same buffer in a diode array stopped-flow spectrophotometer (Applied Photophysics) housed inside an anaerobic chamber (<0.5 ppm O_2_) at 24 °C. Spectra were recorded using a xenon lamp at intervals from 1 msec to 60 or 200 s for wild-type or P1173L MTR. The change in absorbance at 526 nm (Δε_526 nm_ = 8 mM^−1^ cm^−1^) and 590 nm (Δε_526 nm_ = 2.8 mM^−1^ cm^−1^) were each fitted to Equation 3 to obtain a double exponential fit:

(4) $$\Delta A_{526\;\text{nm}/590\;\text{nm}}=A1\;*\;e^{-t*k1}+A2\;*\;e^{-t*k2}$$

where k1 and k2 are the observed rate constants of the two phases and A1 and A2 are the amplitude changes associated with the respective phases.

Conversion of base-off/His-on cob(II)alamin on MTR to base-off cob(II)alamin upon complex formation with ^ΔC^MTRR_hq_ was measured by rapidly mixing MTR•cob(II)alamin (40 μM) with ^ΔC^MTRR_hq_ (70 μM) in Buffer D under anaerobic conditions at 24 °C. Spectra were recorded at intervals from 1 msec to 60 s and the absorbance change at 464 nm was fitted to equation 3.

Formation of MeCbl from the pre-formed MTR•^ΔC^MTRR complex was performed by pre-incubating MTR•cob(II)alamin (40 μM) with ^ΔC^MTRR_hq_ (70 μM) in Buffer D for 1 min in the anaerobic chamber at room temperature. The MTR•MTRR complex was rapidly mixed with AdoMet (400 μM) and spectra were recorded at intervals from 1 msec to 5 s for wild-type and 1 msec to 200 s for P1173L MTR. Absorbance changes at 526 and 590 nm were fitted to equation 4.

Cob(I)alamin bound to MTR was generated by mixing MTR•cob(II)alamin (20 μM) in Buffer D with 125 μM titanium(III)citrate prepared as described previously (38), and incubating at room temperature for 20 min in Buffer D. Methylation of cob(I)alamin and entry of MTR into the catalytic cycle was monitored by rapidly mixing MTR•cob(I)alamin (20 μM) with AdoMet (200 μM) under anaerobic conditions at 24 °C. Spectra were recorded at intervals from 1 msec to 2 s and changes in absorbance at 440 nm and 390 nm were fitted to Equations 4 and 5, respectively:

(5) $$\Delta A_{390\;\text{nm}}=A1\;*\;e^{-t*k1}$$

where k1 is the observed rate constant and A1 is the amplitude change associated with the reaction.

In experiments involving ^ΔC^MTRR_hq_ and P1173L MTR, spectra were monitored for longer durations. To minimize the photobleaching of ^ΔC^MTRR_hq_ and slow photolysis of the MeCbl product, a 320 nm cutoff filter was inserted in the light path immediately following the xenon lamp, and the slit width of the incoming light was adjusted to 0.5 mm.

## Data availability

All data are contained within the manuscript and the supporting information section.

## Supporting information

This article contains supporting information.

## Conflict of interest

RB is a consultant for Zyphore Therapeutics Inc.
