# Supplementary material for: The common homocystinuria-associated P1173L variant of human methionine synthase impairs reductive methylation
Source: J Biol Chem. 2025 Jun 11;301(7):110366. doi: 10.1016/j.jbc.2025.110366 (PMC12272874; doi:10.1016/j.jbc.2025.110366)
Supplement: Supporting information [file mmc1.docx]

**Supporting Information**

The common homocystinuria-associated P1173L variant of human methionine synthase impairs reductive methylation

Arkajit Guha and Ruma Banerjee*

Department of Biological Chemistry, Michigan Medicine, University of Michigan, Ann Arbor, MI 48109

Running title: Clinical variant impairs repair

*Corresponding author. Email: [rbanerje@umich.edu](mailto:rbanerje@umich.edu)

**Table of content**

**Table S1**. Parameters used for EPR simulations

**Figure S1**. Analysis of products formed in the reductive methylation reaction

**Table S1. Parameters used for EPR simulations**


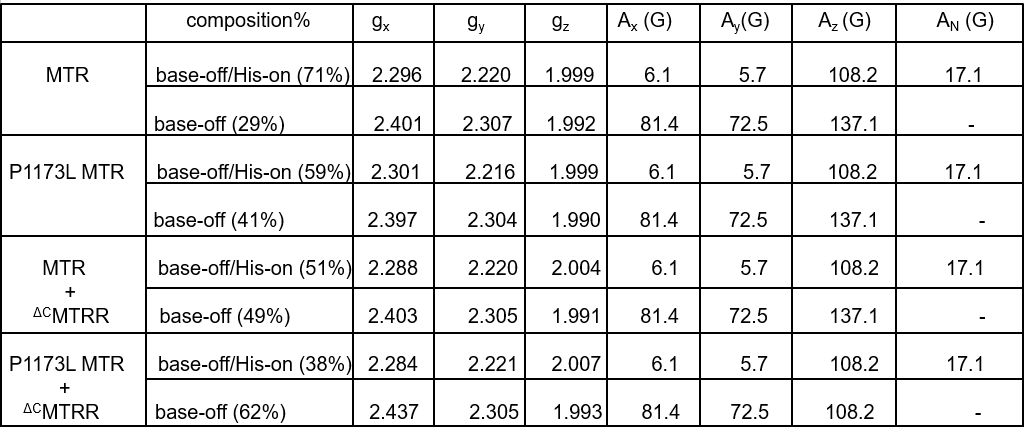

**Figure S1**. **Analysis of products formed in the reductive methylation reaction.** *A-B*, *S*pectra of 25 µM each of cob(II)amin, 5-c MeCbl and 6-c MeCbl in 100 mM potassium phosphate, pH 7.4 (*A)* and oxidized, semiquinone and hydroquinone forms of FMN bound to ^ΔC^MTRR in the same buffer (*B)*. ^ΔC^MTRR purifies predominantly in the oxidized FMN form but a small portion of the enzyme exists in the semiquinone form as seen by the peak at 590 nm. *C,* Formation of MeCbl by wild-type MTR during reductive methylation (Fig. 4B, *black spectrum*) was confirmed by HPLC analysis. *D,* Time-dependent changes in absorbance at 526 nm (*blue*) and 590 nm (*red*) were monitored up to 200 sec to determine the rate constants for reductive methylation by P1173L MTR.
